# Role of extracellular vesicles in osteosarcoma

**DOI:** 10.7150/ijms.74137

**Published:** 2022-07-04

**Authors:** Qifan Yang, Jing Liu, Bo Wu, Xinyu Wang, Ye Jiang, Dong Zhu

**Affiliations:** 1 Department of Orthopaedics, the First Hospital of Jilin University, Changchun, Street Xinmin 71, China.; 2 The first clinical medical college of Bin Zhou Medical College, Street Huanghe 661, China.

**Keywords:** osteosarcoma, extracellular vesicles, immune escape, chemotherapy resistance, microenvironment

## Abstract

Osteosarcoma is a malignant bone tumor characterized by the direct production of osteoid tissue from tumor cells. Extracellular vesicles are membranous vesicles released by cells into the extracellular matrix, which exist widely in various body fluids and cell supernatants, and stably carry some important signaling molecules. They are involved in cell communication, cell migration, angiogenesis and tumor cell growth. Increasing evidence has shown that extracellular vesicles play a significant role in osteosarcoma development, progression, and metastatic process, indicating that extracellular vesicles can be use as biomarker vehicles in the diagnosis and prognosis of osteosarcoma. This review discusses the basic biological characteristics of extracellular vesicles and focuses on their application in osteosarcoma.

## Introduction

**Figure 1:**
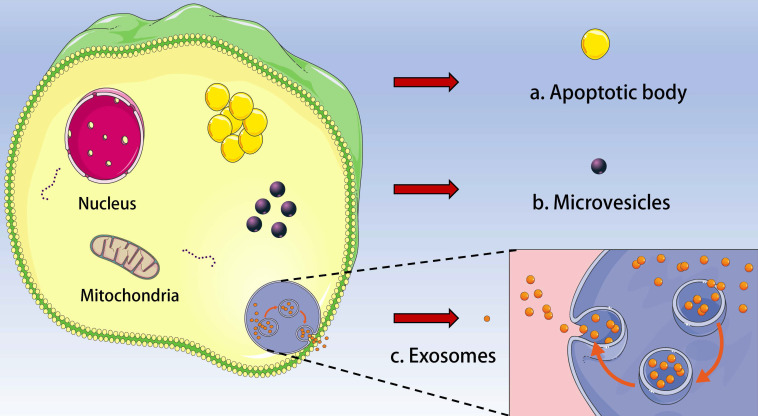
Types of extracellular vesicle (EV). Based on the biogenesis, diameter and biophysical properties, EV can be divided into three subgroups: **a.** Apoptotic body; **b.** Microvesicles and **c.** Exosomes.

Extracellular vesicles (EVs) are a general term for all kinds of vesicle structures with membrane structure released by cells. Based on the biogenesis, diameter and biophysical properties, EVs can be divided into three subgroups: exosomes, microvesicles/ectosomes, and apoptotic body (**Figure 1**). EVs play a critical role in cell communication and body regulation through signal molecules such as proteins and lipids on the membrane and contents (neurotransmitters, enzymes, hormones and nucleic acids) wrapped inside the membrane 1-3. Interestingly, EVs were initially regarded as “cell dust” and a mechanism for processing cellular components, and now EVs were considered as promising circulating biomarkers of diseases 4. In addition, EVs are extensively involved in apoptosis, tumor development, angiogenesis and immune responses 5. Almost all cells can secrete EVs under physiological or pathological conditions, and EVs can also be found in almost all body fluids such as blood, urine, and saliva 6. EVs offer great advantages in cancer research.

Osteosarcoma (OS) is one of the most difficult diseases in the field of orthopedics and is also the focus of current medical research. OS, comprising around 1% of all human malignancies, is a heterogeneous malignant spindle cell tumor characterized by the formation of immature osteoid tissue or osteoid 7, 8. In general, OS occurs mainly in adolescents, but there is a second incidence peak in older adults over 60 years of age 9. The standard treatment for OS mainly includes surgery, post-operative chemotherapy and neoadjuvant chemotherapy. Although the 5-year survival rate of these treatment is 60-70% 10, 11, for patients with metastatic and/or recurrent OS, the original treatment method does not guarantee a favorable outcome 12, 13. Therefore, the major translational objective in OS research is to find new therapeutic markers with great clinical potential. In this review, we mainly discuss the role of EVs in OS, including regulating OS metastasis, tumor microenvironment, immune escape, chemotherapy resistance, which make them to become promising biomarkers and therapeutic targets for OS.

## EVs biogenesis

Exosomes are produced by inward invagination through the endosomal membrane pathway 14. Firstly, the inward budding of cellular plasma membrane promotes the formation of endosomes 15. Vesicles are formed by further inward budding of limiting membranes inside endosomes, causing the formation of the multivesicular body (MVB), which is characterized by the intraluminal vesicles 16, 17. Upon maturation, MVBs may fuse with the plasma membrane and secrete exosomes, or degrade the cargo by lysosomal fusion. During this process, transmembrane proteins, cytosolic contents, and peripheral proteins are intergrated into the invaginating membrane, and then MVBs fuse with the lysosome, resulting in the vesicular contents degradation 18, 19. Similarly, MVBs can fuse with the plasma membrane and release vesicles to the extracellular space in an exocytotic manner 20. The released small membrane-bound lipid vesicles with a diameter ranging from 30 to 200 nm are considered as exosomes.

In addition, EVs can directly bud in the plasma membrane. Microvesicles have extremely heterogeneous sizes ranging from 50 nm to 10 μm 21. The mechanism of microvesicle biogenesis is correlated with non-apoptotic plasma membrane blebs which exist in the highly aggressive cancer cells 22. These vesicles expand and contract on the cell surface to promote cell motility and can be released as microvesicles, which are rearranged by the actin cytoskeleton in the vesicle budding neck, leading to membrane scission 23-25. Tumor cells during migration *in vivo* enable to adopt a phenotype called amoeba, which is related to the blebbing of extensive plasma membrane and microvesicle releasing, indicating that this type of EVs biogenesis plays an important role in tumor invasion and metastasis 25-28.

## EVs cargo

In the tumor microenvironment, the functional properties of EVs depend on their cellular cargo and their metabolic dynamics 29. One of the major challenges in this field is to identify the pro-tumorigenic components of cancer EVs and the pathways that lead to their binding into vesicles. EVs contain various contents as follow.

Protein cargo: Proteins are integrated into EVs by interrelating with components of the EV biogenesis machinery 30, 31. Generally, EVs are very rich in the cytoskeleton protein, the cytoplasmic protein, the cell membrane protein, the heat shock protein, and proteins participating in vesicle transport, however, fewer organelle proteins exist in the cell 32.

DNA cargo: Several evidence has shown that the presence of the mitochondrial DNA, the fragmented genomic DNA and even the parasitic DNA in EVs 33-35. Although the mechanism of DNA incorporation in EVs remains unknown, the fact that EV genomic DNA fragments are evenly distributed throughout the whole genome indicates a random process 36. DNA fragments secreted by EVs prevent the activation of cytoplasmic DNA sensors, thereby promoting cellular homeostasis, which might be particularly critical in cancer because increased levels of DNA damage require efficient cytoplasmic DNA removal by EVs 37.

RNA cargo: EVs contain complete mRNA, fragmented mRNA, long non-coding RNA, ribosomal RNA, and miRNA 9. EVs are loaded with various contents which reflect different states of the parent cell and affect the performance and functions of EVs.

## EVs detection methods

### Microscopy based methods

Microscopy based methods, such as scanning electron microscopy (SEM), transmission electron microscopy (TEM), cryo-electron microscopy (Cryo-EM), and atomic force microscopy (AFM), are widely used in measuring the physical features (size, distribution, concentration, etc.) of EVs. SEM takes images of the EV sample through scanning the surface and provides information on the three-dimensional surface topography and the elemental composition of samples 38. TEM has a high resolution and is capable of imaging objects <1 nm. Unlike SEM and TEM, which require extensive fixation and staining, Cryo-EM is able to analyze EVs in frozen samples because the interference of dehydration and chemical fixatives is excluded 39.

### Nanoparticle tracking analysis

Nanoparticle Tracking Analysis (NTA) is a tracking method for determining particle concentration and size distribution. Nanoparticles in their suspensions undergo irregular Brownian motion due to the impact of surrounding solution molecules 40, 41. According to the Stokes-Einstein equations, there is a quantitative relationship between the speed of movement of these particles per unit time and their own particle size, the viscosity of the solution and the temperature. Therefore, by observing the trajectory of the particles in solution, the particle size data associated with it are derived. At the same time, each observed particle is tracked and analyzed by the instrument's built-in high-speed camera and software, ultimately providing an analysis of the particle size number distribution and particle concentration that differs from that of conventional particle size meters 42.

### Small particle flow cytometry

Conventional flow cytometry has limited resolution in detecting small particles less than 500 nm in diameter 43. The high optical background due to the presence of small particles in sheath fluids is also a problem 44. To address these challenges, highly sensitive flow cytometers, which can discriminate particles of 100 nm in diameter, are under development 45.

## EVs in osteosarcoma

EVs are mediators in the tumor microenvironment and are thought to play a crucial role in communication between tumor cells and other cells. Recent evidence has shown that EVs also closely participated in the tumorigenesis, proliferation, metastasis, immune evasion and chemoresistance of OS, and are regarded as potential biomarkers and therapeutic targets for OS. Understanding the role of EVs in OS is crucial for future treatment and prolonged survival of OS patients. Roles of EVs in OS are thoroughly discussed as follow.

### The role of EVs in metastasis of osteosarcoma

Despite recent advances in the diagnosis and treatment of OS, many patients still had the poor survival rate. It was estimated that 10% to 20% patients develop metastasis before diagnosis, with 5-year overall survival rates (OSR) of less than 20% 46. Understanding the metastasis process is one of the current focuses of OS researches, which could help develop strategies for treating metastatic disease and improving OSR. Interestingly, EVs are considered to play an important role in tumor metastasis, and they participate in the metastasis of tumor cells mainly through three ways 47, 48: tumor cells directly promote metastasis by secreting EVs; EVs influence the microenvironment of tumor cells; EVs indirectly mediate metastasis via transforming distant mesenchymal cells. MiRNA could be transported between tissues by EVs. Bioinformatics researches demonstrated that miRNAs could regulate metastasis through influencing mitogen activated protein kinase 1 (MAPK1), neuroblastoma Ras (NRAS), fibroblast growth factor receptor substrate 2 (FRS2), and Quaking (QKI) 49. MiR-675 from serum EVs was found to be a potential new biomarker for OS metastasis, which also demonstrate that tumor cells could produce EVs to influence their own growth and metastasis 50. MiR-143 could suppress the lung metastasis of OS, and the increasing of miR-143 could promote apoptosis, and inhibit OS cell growth 51, 52. MiR-21 may remarkably influence the phenotype of OS cells, resulting in progression, metastasis, angiogenesis, and immune escape in OS 53. MiR-1307 from OS cells derived EVs induced the proliferation, migration and invasion of OS cells via targeting AGAP1 54. Moreover, BMSCs-derived EVs miR-208a could promote the progression of OS via targeting PDCD4 55. EVs from adipose mesenchymal stem cells (AD-MSCs) could foster the metastasis of OS by increasing the level of COLGALT2 56. BMSCs-derived EVs encapsulated long non-coding PVT1 RNA and transported it to OS cells, promoting tumor growth and metastasis 57. BMSCs-derived EVs LCP1 can induce metastasis via JAK2/STAT3 axis 58. Macrophage-derived EVs lnc-LIFR-AS1 can induce the progression of OS cells via miR-29a/NFIA pathway 59. The fusion protein Rab22a-NeoF1 can induce OS metastasis in lungs via the activation of RhoA 60. In another study, TGF-β1 was found to enhance the level of the proteoglycan by suppressing miR-143, enhancing the metastasis of OS 61. TGF-β expression has been detected in OS cells derived EVs, which participates in tumor invasion, angiogenesis, and metastasis. TGF-β could directly or indirectly influence the production of chemokine ligand 16 (CXCL16), regulating the metastasis of OS cells 62. TGF-β from EVs could also induce the differentiation of monocytes and the accumulation of immature myeloid suppressor cells (MDSCs) 63, 64. TGF-β was also confirmed to be present in the exosome surface, which could induce the production of IL-6 and IL-8 by MSCs, enhancing a pro-inflammatory OS microenvironment favorable for metastasis 65.

EVs have been identified as important factors in regulating tumor and mesenchymal cell induction of metastasis and in regulating oncogenic phenotypes 66, 67. EVs could promote metastasis of OS to local or distant tissues and organs. OS cells could secret transforming EVs, which further confer tumor-like phenotypes on normal recipient cells, promoting metastasis 68. EVs derived from OS cells could mediate fibroblast differentiation to cancer-related stromal fibroblasts (CAFs) phenotype via SMAD2 and TGF-β1 pathway, which show the possibility about OS cells influence distant cells to promote metastasis 69. OS-derived EVs could also induce M2 type macrophages polarization to mediate invasion and distant metastasis of OS cells 70. OS-derived EVs significantly induce mesenchymal stem cells (MSCs) metastasis via IL-6/STAT3 axis 71. Compared with the non-metastatic OS-derived EVs, metastatic OS-derived EVs promoted the migration ability of osteoblasts 50. In addition, the uptake of OS-derived EVs by endothelial cells mediated the production of pro-angiogenic factors, demonstrating EVs could modulate the invasiveness of cells through affecting OS microenvironment 72. Another study found that injection of mesenchymal stem cells (MSCs) co-cultured with metastatic OS-derived EVs could promote tumor growth and metastatic dissemination to distant organs 65. The capacity of stromal cells to influence OS cells has been confirmed in EVs secreted by CAFs. Compared with non-cancer fibroblast, transferring the exosomal cargo from CAFs to tumor cells significantly promoted migration and invasion, which may be attributed to the enrichment of miR-1228 in CAFs 73. COL6A1 could be carried by OS-derived EVs and activate CAFs to promote OS metastasis 74. Altogether, EVs and their contents could educate tumor cells, inducing a pro-metastatic and tumorigenic phenotype, and promote OS metastasis to local or distant tissues.

### EVs and osteosarcoma microenvironments

In the microenvironment of OS, tumor cells, mesenchymal stem cells, immune cells, fibroblasts, osteoclasts, osteoblasts, and endothelial cells coexist and interact with each other, in which EVs play a vital role 5, 75, 76. An important function of extracellular vesicles (EVs) is to communicate with target cells. The finding of extracellular vesicles in osteoblasts and osteoclasts provides a strong theoretical basis for studying the role of extracellular vesicles in the microenvironment of OS 77. Interestingly, tumor cells can also secrete exosomes, which can promote tumor growth, metastasis and angiogenesis by regulating tumor microenvironment 78, 79.

OS is generally considered to be an osteoblastic cell line tumor, and osteoclasts have been shown to play a key role in OS invasiveness and adverse reactions to chemotherapy 80, 81. Osteoclast formation and bone resorption are stimulated by the pro-osteoclastogenic cargo of OS-derived EVs 72, 82. Raimondi et al. found the pro-osteoclastic miRNA cargo in OS-derived EVs, containing miR-148a-3p and miR-21-5p, which are involved in the establishment of tumor microenvironment 72. These suggest that the pro-osteoclastogenic cargo of EVs have a specific role in modifying bone remodeling homeostasis in the OS bone microenvironment.

Potential effects of EVs from OS cells on bone marrow stroma have also been reported. Biomechanical stress in bone marrow stroma can increase intracellular calcium level, accelerate the formation of EVs, and elevate the expression of matrix metalloproteases (MMPs). The nuclear factor kappa-B (NF-κB) receptor activator ligand (RANKL) is regarded as an important factor regulating osteoclast differentiation because it plays an important role in activating MMPs and stimulating osteoclast formation 83. Lim et al. found the nucleic acid transfer from the bone microenvironment to breast cancer cells via EVs 84. Transmembrane 4 superfamily protein CD9 was found in OS tumor microenvironment, which is also a membrane fusion protein of osteoclast precursors and is associated with the regulation of osteoclast differentiation and maturation 62, 85. CD-9 is abundant in EVs, which can regulate osteoclast differentiation. In cancers, overexpression of CD-9 induces osteoclast bone resorption in the bone micro environment 86. Yi et al. 87 also found the regulatory function of CD-9 in MMPs induced cancer migration and invasion. Blocking of CD-9 by KMC8 could inhibit the formation of multinucleated osteoclasts and regulate the differentiation of osteoclasts 88. The expression of transforming growth factor-β (TGF-β) in serum of OS patients was significantly increased, which can stimulate migration of OS cells 89. TGF-β contained in extracellular vesicles can increase the accumulation of immature myeloid cells, which can accelerate osteoclastic bone resorption 64. Thus, inhibiting EVs secretion may improve the bone microenvironment and suppress tumorigenesis.

Mesenchymal stem cells (MSCs) are believed to support tumor progression by vesicles secretion 90. Therefore, there is increasing interest in studying the activity of MSCs derived EVs on tumor cells. EVs secreted by mesenchymal stem cells (MSCs) can change the phenotype of OS cells as regulators in the tumor microenvironment. Communication via EVs of stressed mesenchymal stem cells (SD-MSCs) significantly affects the metastasis potential of OS cells, which is closely related to the miRNA content of EVs 91. EVs from human marrow mesenchymal stem cells (hBMSCs) can act as paracrine factors to activate Hedgehog signaling pathway in OS cells, regulating OS growth 92. EVs from hBMSCs can promote the progression of OS by increasing the expression of HIF-1α and related gene through PI3K/AKT pathway 93. Besides, EVs from hBMSCs can also promote tumorigenesis and metastasis of OS by accelerating oncogenic autophagy 94. However, MSCs are not the only component of OS microenvironment. Further studies of how EVs act on OS cells will help to discover new mechanisms of the cell-to-cell communication in the microenvironment and to identify novel targets.

The EVs from OS cells can also regulate signaling pathways. EVs is widely involved in the regulation of multiple components of the Wnt pathway, which is closely related to OS progression 95. It was discovered that the up-regulation of CD82 and CD9 mediated cells to secrete exosomes, which significantly inhibiting Wnt signaling 96. However, the development of OS involves the interaction of various signaling pathways. Therefore, the in-depth study of their correlation can improve the therapy of OS.

### EVs and immune escape

EVs derived from cancer could promote tumor immune escape through multiple mechanisms 97, 98. Overexpression of PD-L1 is closely associated with metastasis in OS. In order to evade immune surveillance, cancer cells activate the programmed death ligand 1 (PD-L1) pathway 99. On the other hand, malignant tumors released EVs carrying PD-L1, which can be used to predict the efficiency of anti-PD-1treatment 98. EVs secreted by OS have a special cargo, which can mediate differentiation of CD4 + cells into T regulatory phenotype, and result in immune evasion 100. EVs from the serum of dogs diagnosed with OS may help to discover the mechanism of immune evasion in OS. Increased expression of plasma protease C1 inhibitor and decreased expression of C1qa in exosomes of osteosarcoma dogs may prevent the activation of classic pathways as potential escape mechanisms 101. In addition, EVs derived from metastatic OS cells can modulate TAMs signaling, promote M2 phenotype, and create an immunosuppressive, pro-tumor microenvironment 102.

### EVs in chemotherapy resistance of osteosarcoma

Neoadjuvant chemotherapy combined with surgery is the main strategy for OS currently. However, some patients developed resistance to chemotherapy, posing a huge challenge to OS treatment. Recent studies have shown that EVs play a crucial role in multidrug resistance (MDR) of osteosarcoma 103-105, which is a major obstacle to successful therapy and good clinical outcome of OS 106. OS-derived EVs can reduce the sensitivity of OS cells to doxorubicin and induce the MDR phenotype in doxorubicin-sensitive cells by transferring MDR-1 mRNA, suggesting a mechanism by which drug-resistant tumor cells spread drug unresponsiveness to sensitive cells, promoting chemotherapy resistance 105. In addition, EVs induce MDR through the transfer of specific bioactive molecules, such as non-coding RNAs and proteins 107. Moreover, miRNAs from EVs cargo have attracted much attention in this field because they can interfere with gene expression and participate in multiple drug resistance mechanisms 108, 109. Xu et al. found that the level of microRNA and mRNA in exosomes of OS can be used to predict the sensitivity to chemotherapy. They isolated miR-135b and other RNAs that can make OS resistant to chemotherapy drugs from OS cell-derived exosomes 110. Besides, EVs have be reported to induce chemoresistance of OS by transmitting circular RNAs (circRNAs), indicate exosomal circRNAs as new targets to be used for addressing OS chemoresistance 111. Researchers also used OS animal models and patient samples to investigate the role of protein cargos, showing that exosomes exhibited unique protein signatures associated with drug resistance 112. These studies explain part of the mechanism of drug resistance in OS cells and provide a new reference for the selection of drugs in OS patients. Therefore, isolating EVs and analyzing of their cargo may reveal new biomarkers that can be used to improve therapeutic efficacy. However, there are still some limitations to the clinical application of exosomes 113. Subsequent study should seek to improve their production and storage processes to prevent loss of function and ensure the safety of EVs treatment.

### EVs as biomarkers for diagnosis and prognosis in osteosarcoma

EVs have attracted extensive attention due to their possible roles in early diagnosis, prognosis prediction, and efficacy assessment of OS (**Table 1**). They can be easily obtained from body fluids, and their cargo is inside a membranous structure, which provides long-term storage stability prior analysis, making them viable for clinical application in the diagnosis and prognosis of OS 114, 115. Nowadays, more and more researchers are using a variety of strategies to screen EVs markers, which can reflect the physiological and pathological state of cells. PD-L1 and exosomal N-cadherin detected from the serum of patients with OS can predict the progression of pulmonary metastasis 116. SENP1 derived from EVs can be used as a novel prognostic biomarker in OS patients 117. The excess of EVs related DNAs of repetitive elements suggests that they may serve as biomarkers for OS 118. Moreover, CASC15 overproduction was discovered in EVs form the plasma of patients with OS, as well as the OS tissues and cell lines 119.

The RNAs in EVs cargo can also be used as effective biomarkers in the diagnosis and progression of OS. Elevated tumor mutation burden in RNA sequences from metastatic EVs plasma samples was observed in a pilot study 120. The membrane of exosomes can make miRNA in exosomes relatively stable, thus increasing the feasibility of clinical application of miRNA in early diagnosis of OS 121-123. Serum exosome miRNAs were regarded as a promising diagnostic biomarker that can distinguish differences in drug resistance in OS 110. Another study found that lung metastasis resulted in significantly elevated expression of circulating EVs-derived miR-675, which may be used as a novel biomarker for OS metastasis in the future 50. The level of exosomal miR-25-3p were also remarkably associated with poor prognosis of OS 109. EVs derived miR-101 is considered as a possible circulating biomarker of OS metastasis 124. Besides, several new miRNAs were found in OS cells and related EVs, indicating these miRNAs may possibly be applied as biomarkers for OS 125. The upregulation of miR-21-5p and miR143-3p in the EVs of metastatic osteosarcoma cells also suggests their potential to become prognostic biomarkers for OS 49. Through high-throughput sequencing, Ye et al. found exosomal miRNAs with different expression in OS and healthy controls, suggesting their possibility as new diagnostic biomarkers 126. More recently, Zhang et al. demonstrated that exosome derived miR-101 could become a hopeful circulating biomarker of osteosarcoma metastasis 124.

Additionally, EVs derived lncRNAs and circRNAs are also considered as promising biomarkers for OS, whose expression is correlated with diagnosis, prognosis and metastasis of osteosarcoma 127-132. Li et al. found a positive feedback loop between EVs derived linc00852 and AXL, suggesting its potential to be a new OS biomarker 133. Another study discovered that the levels of hsa-circ-103801 were elevated in serum of OS patients who had poor prognosis, demonstrating hsa-circ-103801 could be used as prognostic biomarker for OS 111.

EVs derived proteins are also regraded as promising biomarkers for predicting prognosis in OS patients. In terms of EVs-related proteins, circulating EVs-related TGF-β levels were significantly elevated in patients with OS 65. EVs-associated proteins were also useful to distinguish serum of OS from serum of healthy animals 101. Wang et al. obtained PD-L1 and N-cadherin from the serum of OS patients as biomarkers to predict OS metastasis 116. Collectively, EVs derived cargoes have a promising prospect to be applied as new diagnostic and prognostic biomarkers for OS. However, there are still several challenges that need to be considered before clinical diagnostic applications of EVs 134. Scholars need to focus on standardizing and improving EVs separation methods, as well as standardizing pre-analysis variables to ensure the reliably assess of EVs.

### EVs in the treatment of osteosarcoma

The ability to exchange information and to deliver bioactive substances to target cells gives EVs great potential for treating human disease 135. EVs can be transferred to target organs, and thus are widely considered as natural nanocarriers, which show great prospect for application as drug targeting vectors 136-142. These researches opened a new area in OS research, linking emerging nanocarrier EVs to the progression, prognosis, and treatment of OS.

EVs derived RNAs may be used as therapeutic targets in OS. Several dysregulated EVs-derived miRNAs were discovered in patients with OS 126. EVs derived from cisplatin-resistant OS cells could transfer the resistance to the recipient cells and inhibit apoptosis, which is closely associated with the expression of exosomal hsa_circ_103801 111. EVs-derived miR-206 could suppress OS progression through inhibiting the proliferation, migration and invasion of OS cells via targeting TRA2B 138. EVs-derived miR-101 has been found to suppress metastasis in OS 124. In another study, EVs-derived miR-1228 was found to facilitate migration and invasion of OS through inhibiting the level of SCAI in OS 73. EVs-derived lncRNA LIFR-AS1 could mediate OS progression via miR-29a/NFIA axis 59. However, upregulation of AGAP1 can suppress the function of EVs-derived miR-1307 in OS 54. The application of EVs to encapsulate miRNAs has manifested advantages in the therapy of OS. Artificial miR-143 was introduced into BMSCs and encapsulated in EVs to reduce the metastasis capability of OS cells, and the exosome miRNA transport was better than other methods in intercellular transport 143.

In addition, EVs-derived proteins are also considered as potential targets for treating OS patients. OS-derived EVs were found to induce M2 type macrophages polarization by regulating Tim-3 level 70. Knockdown of CASC15, which is elevated in OS derived EVs, could suppress OS progression through regulating miR-338-3p/RAB14 axis 119. BMSCs-derived exosomal LCP1 mediates OS progression through the JAK2/STAT3 axis, while miR-135a-5p could inhibit tumorigenesis of OS induced by LCP1 58. Heterologous exosomes secreted by MSCs are regarded as reliable source of therapeutic exosomes 144, 145. Combined use of TGF-β inhibitors and IL-6 blockers can reduce drug resistance and prevent the progression of osteosarcoma, which is based on the finding that EVs carry functional TGF-β molecules and elevate IL-6 production, promoting OS growth and metastasis 65.

Researchers have also explored the immune therapeutic effects of EVs in OS. EVs secreted from dendritic cells (DCs) had functional MHC, T-cell and costimulatory molecules, implying that DC vaccination secreted antibodies induce regulatory molecules could improve the immunotherapeutic efficacy in OS 146. Kawano et al. discovered that the combination of DCs and anti-TGF-β antibody could inhibit the proliferation of OS cells, activate the systemic immune system, and improve the treatment efficacy of OS 147. Another study discovered that doxorubicin-loaded exosomes derived from MSCs may be an excellent agent for OS in the future, considering the tumor-targeting function of BM-MSCs 148. All of these studies demonstrate that these targets are promising as potential therapeutic approaches for OS, which may contribute to improve survival of OS patients.

## Conclusions

**Figure 2:**
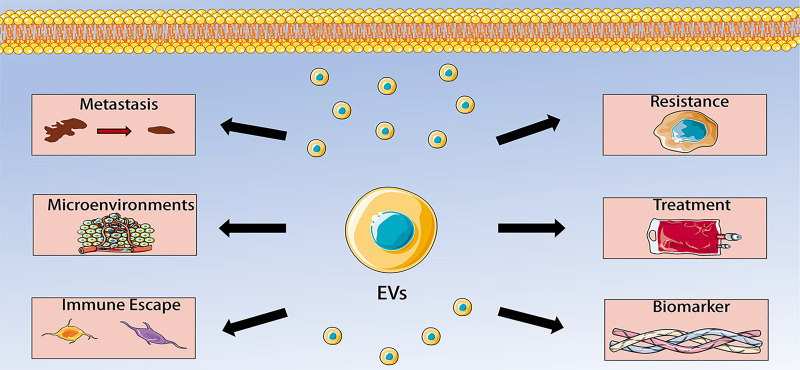
Extracellular vesicles (EVs) in osteosarcoma (OS). The role of EVs in OS includes regulating OS metastasis, tumor microenvironment, immune escape, chemotherapy resistance, which make them to become promising biomarkers and therapeutic targets for OS.

Extracellular vesicles have become the focus of research because of their intercellular communication functions. More and more studies have found that EVs can regulate tumor microenvironment, thereby influencing the occurrence, development, metastasis, immune escape and chemotherapy resistance of malignant tumors. OS has a complex tumor microenvironment in which tumor cells, stem cells, mesenchymal cells, immune cells, fibroblasts and endothelial cells communicate with each other, leading to OS progression. In this communication, extracellular vesicles play a key role. EVs are secreted by a variety of cells, such as osteosarcoma cells, BMSCs, ADSCs, CAFs, and macrophages. The EVs contain multiple kinds of cargoes, including miRNAs, lncRNAs, circRNAs, and proteins. EVs and their cargoes could regulate the activity of recipient cells, including angiogenesis, proliferation, invasion, migration, metastasis and chemoresistance. Moreover, the detection of serum EVs in patients with OS have great value of early diagnosis and prognosis of OS. EVs can also be applied as carriers to transfer therapy agents to targeted sites because they can avoid immune response. Collectively, data from recent studies reveal multiple functions of EVs in OS, including regulating OS metastasis, tumor microenvironment, immune escape, chemotherapy resistance, which make them to become promising biomarkers and therapeutic targets for OS (**Figure 2**). However, the specific mechanisms by which EVs participate in these processes have not been completely elucidated. Therefore, more studies are needed to determine the precise roles of EVs in the pathogenesis of OS. In addition, future studies should also advance EVs purification, characterization, storage and drug loading techniques, which could help to better detect EVs, understand their biological characteristics, and promote EVs into clinical practice. Another issue waiting to be addressed is identifying the exact content of EVs and their safety. There is no doubt that these problems have to be addressed before they can be used in the clinical practice of OS.

## Figures and Tables

**Table 1:** EVs as biomarkers for diagnosis and prognosis in osteosarcoma

Cargotype	Exosomal cargo	Source	Extraction method	Identification method	Clinical value	References
miRNA	miR-675	Serum	Ultracentrifugation	Transmission electron microscope and western blot	Biomarker for predicting OS metastasis	50
	miR-25-3p	Extracellular fluid	Ultracentrifugation	Scanning electron microscope and western blot	Diagnostic biomarkers to indicate poor prognosis of OS	109
	miR-101	Plasma	Differential centrifugation	Scanning confocal microscope and western blot	Circulating biomarkers for OS detection	124
	miR-21-5p	Extracellular fluid	Ultracentrifugation	Nanoparticle tracking analysis	Diagnostic biomarkers for OS	49
	miR143-3p	Extracellular fluid	Ultracentrifugation	Nanoparticle tracking analysis	Diagnostic biomarkers for OS	49
IncRNA	linc00852	Extracellular fluid	Differential centrifugation	Transmission electron microscope and western blot	Biomarkers for OS	133
circRNA	hsa-circ-103801	Serum	Ultracentrifugation	Transmission electron microscope and western blot	Prognostic biomarkers for OS	111
Protein	PD-L1	Serum	Differential centrifugation	Transmission electron microscope, qRT-PCR, nanoparticle tracking analysis	Biomarkers to predict OS metastasis	116
	N-cadherin	Serum	Differential centrifugation	Transmission electron microscope, qRT-PCR, nanoparticle tracking analysis	biomarkers to predict OS metastasis	116
